# Trimetazidine Attenuates Heart Failure by Improving Myocardial Metabolism via AMPK

**DOI:** 10.3389/fphar.2021.707399

**Published:** 2021-09-15

**Authors:** Hongyang Shu, Weijian Hang, Yizhong Peng, Jiali Nie, Lujin Wu, Wenjun Zhang, Dao Wen Wang, Ning Zhou

**Affiliations:** ^1^ Division of Cardiology, Department of Internal Medicine, Tongji Hospital, Tongji Medical College, Huazhong University of Science and Technology, Wuhan, China; ^2^ Hubei Key Laboratory of Genetics and Molecular Mechanism of Cardiologic Disorders, Huazhong University of Science and Technology, Wuhan, China; ^3^ Department of Orthopaedics, Union Hospital, Tongji Medical College, Huazhong University of Science and Technology, Wuhan, China

**Keywords:** pressure overload, heart failure, myocardial metabolism, AMPK, trimetazidine

## Abstract

Energic deficiency of cardiomyocytes is a dominant cause of heart failure. An antianginal agent, trimetazidine improves the myocardial energetic supply. We presumed that trimetazidine protects the cardiomyocytes from the pressure overload-induced heart failure through improving the myocardial metabolism. C57BL/6 mice were subjected to transverse aortic constriction (TAC). After 4 weeks of TAC, heart failure was observed in mice manifested by an increased left ventricular (LV) chamber dimension, an impaired LV ejection fraction evaluated by echocardiography analysis, which were significantly restrained by the treatment of trimetazidine. Trimetazidine restored the mitochondrial morphology and function tested by cardiac transmission electron microscope and mitochondrial dynamic proteins analysis. Positron emission tomography showed that trimetazidine significantly elevated the glucose uptake in TAC mouse heart. Trimetazidine restrained the impairments of the insulin signaling in TAC mice and promoted the translocation of glucose transporter type IV (GLUT4) from the storage vesicle to membrane. However, these cardioprotective effects of trimetazidine in TAC mice were notably abolished by compound C (C.C), a specific AMPK inhibitor. The enlargement of neonatal rat cardiomyocyte induced by mechanical stretch, together with the increased expression of hypertrophy-associated proteins, mitochondria deformation and dysfunction were significantly ameliorated by trimetazidine. Trimetazidine enhanced the isolated cardiomyocyte glucose uptake *in vitro*. These benefits brought by trimetazidine were also removed with the presence of C.C. In conclusion, trimetazidine attenuated pressure overload-induced heart failure through improving myocardial mitochondrial function and glucose uptake via AMPK.

## Introduction

Chronic heart failure (CHF), which attacks over 26 million people worldwide, remains to be a major cause of mobility and mortality for the elderly people (Savarese and Lund, 2017). Although various pathological conditions lead to CHF, hypertension was undoubtedly the most important disease which should be blamed for the large amount of CHF population (Worldwide trends in blood pressure from 1975 to 2015: a pooled analysis of 1,479 population-based measurement studies with 19·1 million participants., 2017). Hypertension, characterized by the left ventricular (LV) pressure overload and the accompanied neuroendocrine disorders, induces cardiac hypertrophy and arrhythmias, eventually leads to heart failure (Zhou et al., 2017; Forrester et al., 2018). Despite intensive research efforts over the past decades, the effective therapeutic targets of hypertensive heart failure remain unclear. Therefore, it is necessary to identify novel mechanism involved in the pathogenesis of cardiac hypertrophy and its transition to heart failure in hypertensive patients.

Increasing evidences suggested that the cardiac metabolic abnormalities and the resultant myocardial starvation and dysfunction were the common pathway leading to heart failure (Knowlton et al., 2014; Riehle and Abel, 2016). Physiologically, the dominant resource of myocardial metabolic substrates is free fatty acid (FFA) instead of glucose. However, glucose replaced the FFA to function as the main substrates for energy generation in pathological states (Fukushima et al., 2015), which even occurred in the very early stage of pressure overload-induced heart failure (Zhang et al., 2013). Worse still, myocardial insulin sensitivity was impaired in failing heart that further deteriorated the myocardial starvation. Therefore, to relieve the myocardial insulin resistance raised as a promising novel approach for the treatment of heart failure (Neubauer, 2007; Knowlton et al., 2014; Fukushima et al., 2015).

Trimetazidine (1-[2,3,4-trimethoxybenzyl] piperazine dihydrochloride), a widely used antianginal agent, has been shown beneficial effects in the treatment of CHF patients (Grajek and Michalak, 2015; Ferrari et al., 2018), despite of the undetermined mechanism. Trimetazidine shifted energy production from fatty acid oxidation to glucose oxidation and increased the activity of pyruvate dehydrogenase (PDH) to facilitate the transformation of pyruvate to acetyl Co-A (Kantor et al., 2000). Trimetazidine was found to activate AMP-activated protein kinase (AMPK), a so-called energy sensor highly conserved master regulator of metabolism at both basal and ischemia/reperfusion (I/R) condition (Liu et al., 2016; Day et al., 2017). AMPK was proved to function as a central modulating factor in the myocardial glucose metabolism (Day et al., 2017).

In the present study, we generated a series of *in vivo* and *in vitro* pathological models in mice and neonatal rat cardiomyocytes (NRCMs) to mimic the progressive development of cardiac dysfunction caused by pressure overload. We tested our hypothesis that trimetazidine ameliorates pressure overload-induced heart failure by improving myocardial mitochondrial function and insulin sensitivity via AMPK. Our studies revealed a novel mechanism of the cardioprotection brought by trimetazidine and unraveled a potential indication of trimetazidine in the treatment of hypertensive heart disease.

## Materials and Methods

### Animal Model

Male C57BL/6 mice were housed in the specific pathogen-free animal center of Tongji Medical Collage, Huazhong University of Science and Technology (HUST). TAC or sham surgery were conducted in 8–10 weeks-old mice as described previously (Goldenberg et al., 2019). All mice were intragastric fed by PBS (1 M, 100 ul) or trimetazidine (20 mg/kg/day, 100 ul) for another 4 weeks since the day of surgery. All procedures were approved by the Experimental Animal Research Committee of Tongji Medical College, HUST and performed according to the NIH guidelines (Guide for the Care and Use of Laboratory Animals, revised 2011).

### Transverse Aortic Constriction (TAC)

TAC surgery was performed in 10-week-old male C57BL/6J mice. The operation process was as follows: The mice were anesthetized by 2% isoflurane and fixed on the operating table. And then they were ventilated artificially with a special mouse ventilator with a tidal volume of 2–3 ml/h and a respiratory rate of 90–110 times/min. After the mouse chest wall hair and skin of the sternum body were cut off, the second rib was exposed, and the mediastinum tissue was bluntly separated to show the surgical field of vision. The mouse aorta was hooked out with a self-made hook to separate the paravascular tissues, and a 27G needle was placed between the aorta and the ligature. After ligation, the needle was withdrawn, the chest cavity was closed and sutured layer by layer, and the tracheal intubation was pulled out after disinfection. Mice were placed on a heating pad for resuscitation, During the resuscitation period, mice were injected with buprenorphine (0.1 mg/kg) intraperitoneally for analgesia. Mice subjected to a sham surgery underwent the same procedure but without constricting the aorta.

### Positron Emission Tomography (PET)

PET imaging was applied to visualize FDG (flurodeoxyglucose) intake of myocardium to evaluate mice myocardial impairment extent. Before PET, the mouse ear tags were removed and corresponding marks on the tail were made. Mice were then injected intravenously with 200 ± 10 μCi [18F]-FDG under the gas anesthesia (2% isoflurane). After 60 min, images were taken by using TransPETBioCaliburn LH systems (RAYDATA, Wuhan, China) and quantified by analyzing the static uptake kinetics up to 20 min. Uptake coloboma on the final images was indicated as the impaired myocardium and identified as region of interest (ROI). Data were presented as standardized uptake value (SUV) calculated from ROI by normalizing the dose and weight of the animal involved in the PET analysis (SUV = activity * weight/injected dose).

### Echocardiography

The mouse echocardiography was performed under anesthesia with 2% isoflurane by using a Visual Sonics Vevo1100 ultrasound machine with MS400 mouse electronic linear array probe (30 Mhz). During the echocardiography, the flow rate was adjusted according to heart rate to maintain it between 500 and 600BPM. The B-Mode images of the parasternal left ventricular long axis and the horizontal short axis of the mitral valve chordae were recorded. Cardiac structural and functional parameters included left ventricular anterior wall end-diastolic thickness (LVAWd), left ventricular internal end-diastolic thickness (LVIDd), left ventricular posterior wall end-diastolic thickness (LVPWd) and left ventricular ejection fraction (LVEF) were collected for at least five cardiac cycles. Each parameter was averaged by at least three biological replicates by two independent subject-blinded researchers.

### Transmission Electron Microscope (TEM)

After sacrifice, the mouse heart tissue was diced into 2 mm thickness, pre-fixed in 3% glutaraldehyde for 2 h and washed by phosphate buffer for three times. Samples were then fixed in 1% osmic acid fixative solution for 1 h to produce osmium black. Samples were washed by phosphate buffer and then dehydrated through a graded series (50, 70, 80, 90% for 10 min, 100% for 15 min) of acetone and embedded in Epon/SPURR resin that was polymerized at 37, 45, 60°C (each temperature for 24 h). Heart tissues were sectioned by 70 nm with a diamond knife on a Leica UC7 ultra-microtome and stained with uranyl acetate (30 min). Sections were washed by double distilled water for three to five times followed by 0.2% lead citrate staining (10 min). Images were taken by an electron microscope (Tecnai G^2^ 20 TWIN, United States).

### Histological Analysis and Immunofluorescence Staining

Heart tissues were fixed with 4% paraformaldehyde, embedded in paraffin and then sectioned at thickness of 4 μm. After routine deparaffinization and hydration, the cardiac paraffin sections were soaked in 3% hydrogen peroxide solution and citric acid antigen retrieval solution for antigen retrieval, 0.25% TritonX-100 and fetal donkey serum were applied for cell membrane permeabilizing and sealing, respectively. Hematoxylin & eosin (HE) and FITC-conjugated wheat germ agglutinin (WGA) staining were used to demonstrate the cross-sectional area (CSA). Masson staining was used to show the collagen deposition. Cell immunofluorescence staining was conducted as previously described (Lu et al., 2021). Images were captured by an Olympus microscope and the cell size was analyzed by Image J software. All data were calculated by Image J-Pro Plus 6.0 software (Media Cybemetics, Bethesda, United States).

### NRCMs Isolation, Cultivation and Treatment

NRCMs were isolated from 1 to 3 days-old SD rat hearts as described previously (Peng et al., 2020). After the heart were taken out, the atrium tissue was removed, and the ventricular tissue was preserved. Then heart tissue was repeatedly washed with pre-cooled PBS, and cut into pieces. After adding 0.125% pancreatin solution, the heart pieces were digested in a 37°C water bath with stirring for seven to eight times (5–8 min each time), and then the digestion was terminated with serum. The supernatant was removed by centrifugation, and 5 ml DMEM was added to resuspended and pipetted for forming a single cell suspension. After repeating the above digestion and centrifugation steps seven to eight times, all cell suspensions were transferred to a culture dish and placed in a cell culture incubator for 2 h to purify cardiomyocytes by the differential adhesion method. The NRCMs were pretreated with trimetazidine (1 uM) for 30 min, and then mechanically stretched to 120% for 24 h. NRCMs were collected for RNA and protein extraction or prepared for immunofluorescence and 2-NBDG uptake assay.

### Adult Cardiomyocyte Isolation

The cardiomyocytes were isolated from adult C57/BL mice heart using the langendorff system as described previously (Torregroza et al., 2021). Briefly, before intraperitoneal injection of 100 mg/kg pentobarbital, mice were injected with 150 units of heparin to prevent blood from clotting in the heart cavity. After the chest cavity was opened, the heart was quickly taken out, and the heart was mounted on the modified Langendorff perfusion system through the ascending aorta. Then the heart was perfused with 37°C oxygen-enriched Krebs-Henseleit buffer (d-Glucose 2 g/L, magnesium sulfate 0.141 g/L, potassium phosphate monobasic 0.16 g/L, potassium chloride 0.35 g/L, sodium chloride 6.9 g/L), followed by 100 U/ml collagenase II (Worthington, LS004176). When the heart started to soften and whiten, the perfusion was stopped, and the heart was cut into small pieces in a petri dish containing the enzyme solution, and gently stirred for further digestion. After collecting the rod-shaped adult cardiomyocytes, CaCl_2_ was gradually added, and finally the cardiomyocytes were plated in a six-well plate pre-coated with type I rat tail collagen for further cultivation. The microscopy was applied to observe cardiac myocytes, if there were many rod-like cells, and then the digestion was stopped by using Dulbecco’s modified Eagle’s medium (DMEM) containing 10% fetal bovine serum (FBS).

### 2-NBDG Uptake Assay

2-NBDG uptake assay was performed as previously described (Geddo et al., 2021). Briefly, isolated adult cardiomyocytes (5 × 10^4^) were seeded in black 96-well culture plates with 100 ul medium each well. After about 2 h, cells were completely adhered. Then cells were incubated with fresh medium containing 10 uM 2-NBDG (Invitrogen, N13195, United States) at 37°C with 5% CO_2_ for 30 min, followed by washing each well with pre-cold PBS triply. Cells were subjected to micro-plate reader by the following parameters: λex/λem = 475/512 nm. Finally, 10 μl of CCK-8 was added to each well and incubated for 4 h, then cells were subjected to a microplate reader with absorbance of 510 nm. CCK-8 was applied to adjust the error of fluorescence intensity of each well caused by the difference in the number of cells.

### Measurements of Cardiac Cholesterol/Triglyceride Levels

The TC and TG are measured using Total cholesterol/Triglyceride assay kit (Njjcbio, China) as described previously. Briefly, 450 ul PBS was added to every 50 mg myocardial tissue. The supernatant was obtained by centrifugation after mechanical homogenization. Then 5 ul supernatant or ddH2O or standard was added to 250 ul TC or TG working solution. After incubating at 37°C for 10 min, the absorbance was measured on a microplate reader. Meanwhile, the protein concentration of the corresponding well is determined by BCA assay. Finally, the concentration was calculated according to the following formula.

$$TC/TG\left(mM\right)=\frac{A_{sample}-A_{blank}}{A_{standard}-A_{blank}}\times C_{standard}÷Cpr$$

### RNA Collection and Real-Time Quantitative PCR

Total RNA was extracted from heart tissues in mice and NRCMs by using Trizol reagent (Life Technologies Inc., Carlsbad, CA, United States) as described previously. Briefly, 1 ml Trizol is added to each well of 6-well plate or 50 mg myocardial tissue, followed by chloroform. With the addition of isopropanol, RNA is extracted. 75% ethanol (solvent: DEPC water) was then applied to wash RNA twice. The obtained RNA with a purity (260/280 ratio) of 1.8–2.0 is reverse transcribed into cDNA (HiScript^®^ II 1st Strand cDNA Synthesis Kit, R211-01, Vazyme, China) according to the manufacturer’s instructions. All samples were processed in a triplicate manner and a melting curve was also conducted to assure the specificity. GAPDH was used as a housekeeper gene to normalize the results. The specific primers are listed in Supplementary Table S1.

### Western Blot

Total protein was extracted from heart tissue using RIPA buffer (Beyondtime, P0013, China) as described previously (Zhou et al., 2020). Briefly, two to three grinding beads with a diameter of 3–4 mm and 1 ml RIPA buffer added with protease inhibitors (cOmplete™,04693116001, Roche) and phosphatase inhibitors (phosSTOP™,04906845001, Roche) were added to the 50 mg tissue sample, and grind for two cycles at 60 Hz (work for 60 s, stop for 60 s). Membrane protein and cytoplasm protein was separated by using Membrane and Cytosol Protein Extraction Kit (Beyotime, P0033, China) according to the manufacturer’s instruction. Briefly, 1 ml of RIPA with protease inhibitor was added into 100 mg of tissue, then the cells were fully disrupted by a glass homogenizer. After removing the nucleus and unbroken cells by centrifugation, cytoplasmic protein was collected. Finally, the cell membrane extraction reagent is added to extract the membrane protein. The obtained protein was accessed by BCA assay to measure the protein concentration. Equal amount samples were separated by SDS-PAGE and converted to PVDF films. Detected specific proteins by using antibodies, antibodies used in the present study were listed in Supplementary Table S2.

### Statistical Analysis

Data are shown as mean ± SEM. In the statistical analysis, D’Agostino and Pearson omnibus normality test was used to test the normality of the data. If the dataset does not show a normal distribution, Mann-Whitney test and Kruskal-Wallis test will be applied for comparisons of two groups and more than two groups, respectively. Comparison between two groups was performed by unpaired student’s *t* test. Multiple group comparison was performed by two-way ANOVA followed by Turkey procedure for comparison of means. All data analyses were conducted in GraphPad Prism 8.0, as well as the graph generation. A value of *p* < 0.05 was considered as statistically significant.

## Results

### Trimetazidine Ameliorated Pressure Overload-Induced Heart Failure

**FIGURE 1:**
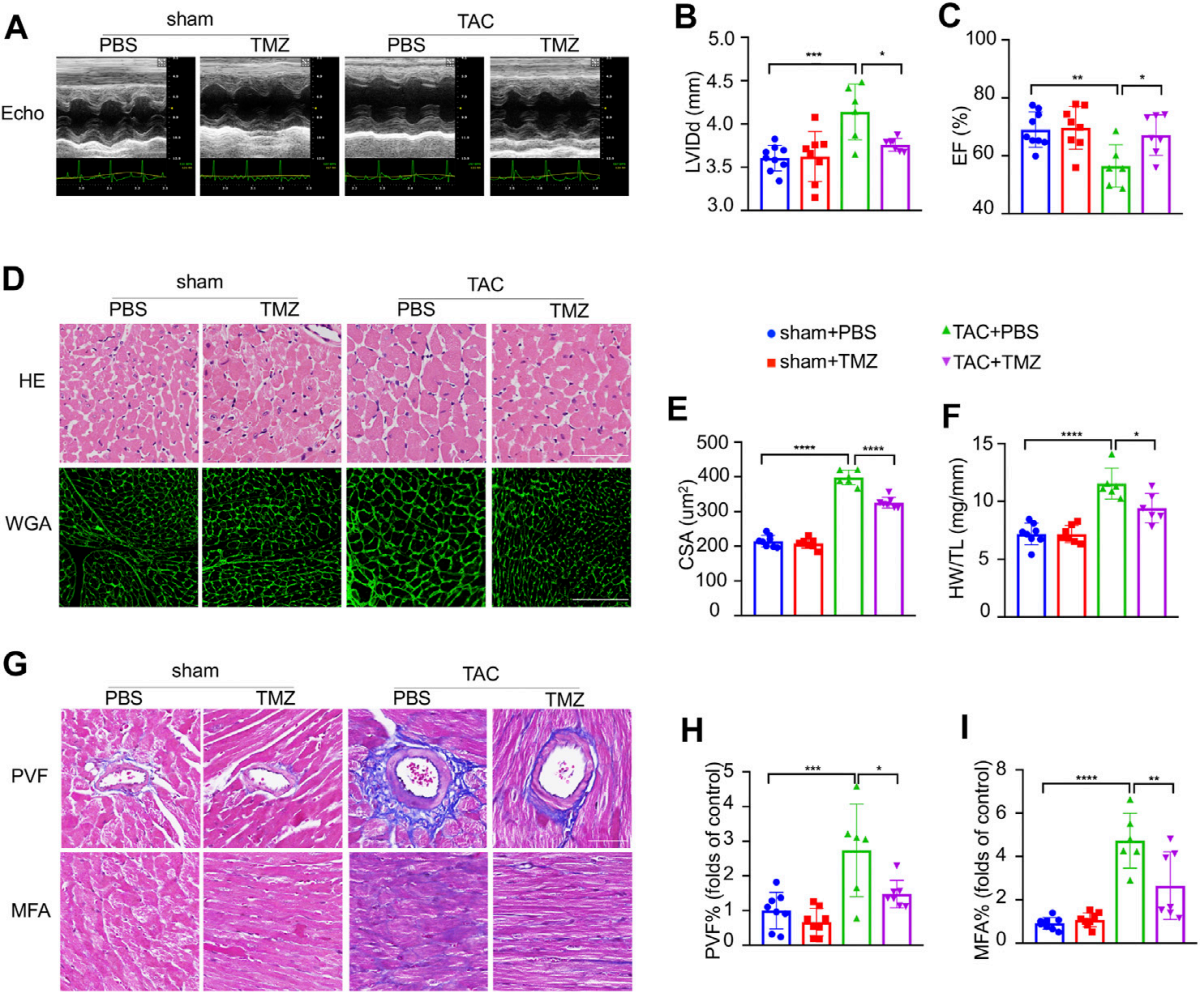
Trimetazidine ameliorated pressure overload-induced heart failure. **(A)** Representative M-mode echocardiograms after transverse aortic constriction (TAC) or trimetazidine (TMZ) treatment. **(B)** Diastole for left ventricular internal diameter (LVIDd). **(C)** Left ventricular ejection fraction (EF). **(D)** Representative histological images of H&E (scale bar = 100 um) and wheat germ agglutinin (WGA) staining (scale bar = 100 um). **(F)** Left ventricular weight (LVW) to tibia length (TL) ratio. **(E)** Relative across sectional area (CSA) of cardiomyocytes calculated from WGA staining. **(G)** Representative cardiac section stained with Masson’s trichrome (scale bar = 100 um). **(H,I)** Quantitative results of perivascular fibrosis area (PVF) and myocardial fibrosis area (MFA). Graphical data are shown as mean ± SEM. One-way ANOVA was used for analysis. *p < 0.05, **p < 0.01, ***p < 0.001, ****p < 0.0001, vs indicated group.

To clarify whether trimetazidine prevents pathological cardiac hypertrophy and heart failure against pressure overload, C57BL/6 mice were subjected to TAC or sham surgery for 4 weeks. Mice developed maladaptive cardiac hypertrophy, represented by a significant increase in left ventricular internal end-diastolic dimensions (LVIDd, sham + PBS vs TAC + PBS, *p* < 0.001, Figure 1A,B; Supplementary Table S3) and impairment of cardiac ejection function (EF, sham + PBS vs TAC + PBS, *p* = 0.002, Figure 1C; Supplementary Table S3) 4 weeks after TAC compared to sham control, which were notably blunted by the treatment of trimetazidine (LVIDd, TAC + PBS vs TAC + TMZ, *p* = 0.032. EF, TAC + PBS vs TAC + TMZ, *p* = 0.011, Figure 1A–C). Compared to sham controls, TAC mice exhibited a significant increase in left ventricular weight (the LVW/TL ratio, sham + PBS vs TAC + PBS, *p* < 0.001) (Figure 1F), and cross-sectional area (CSA) of cardiomyocytes (CSA, sham + PBS vs TAC + PBS, *p* < 0.0001, Figure 1D,E), whereas trimetazidine-treated TAC mice showed relieved hypertrophy in these direct measurements vs PBS-treated TAC mice (Figure 1D,F). Moreover, histological analysis revealed that the trimetazidine-treated TAC mice exhibited remarkably less cardiac fibrosis compared to PBS-treated TAC mice, evidenced by decreased myocardial interstitial fibrosis area (MFA) and perivascular fibrosis area (PFA) (MFA, TAC + PBS vs TAC + TMZ, *p* = 0.023. PFA, TAC + PBS vs TAC + TMZ, *p* = 0.0153, Figure 1G–I).

These *ex vivo* data, consistent with the observations *in vivo*, further supported the protective effects on TAC-induced maladaptive cardiac hypertrophy led by trimetazidine.

### Trimetazidine Improved the Pressure Overload-Induced Impairment of Mitochondrial Structure and Function

**FIGURE 2:**
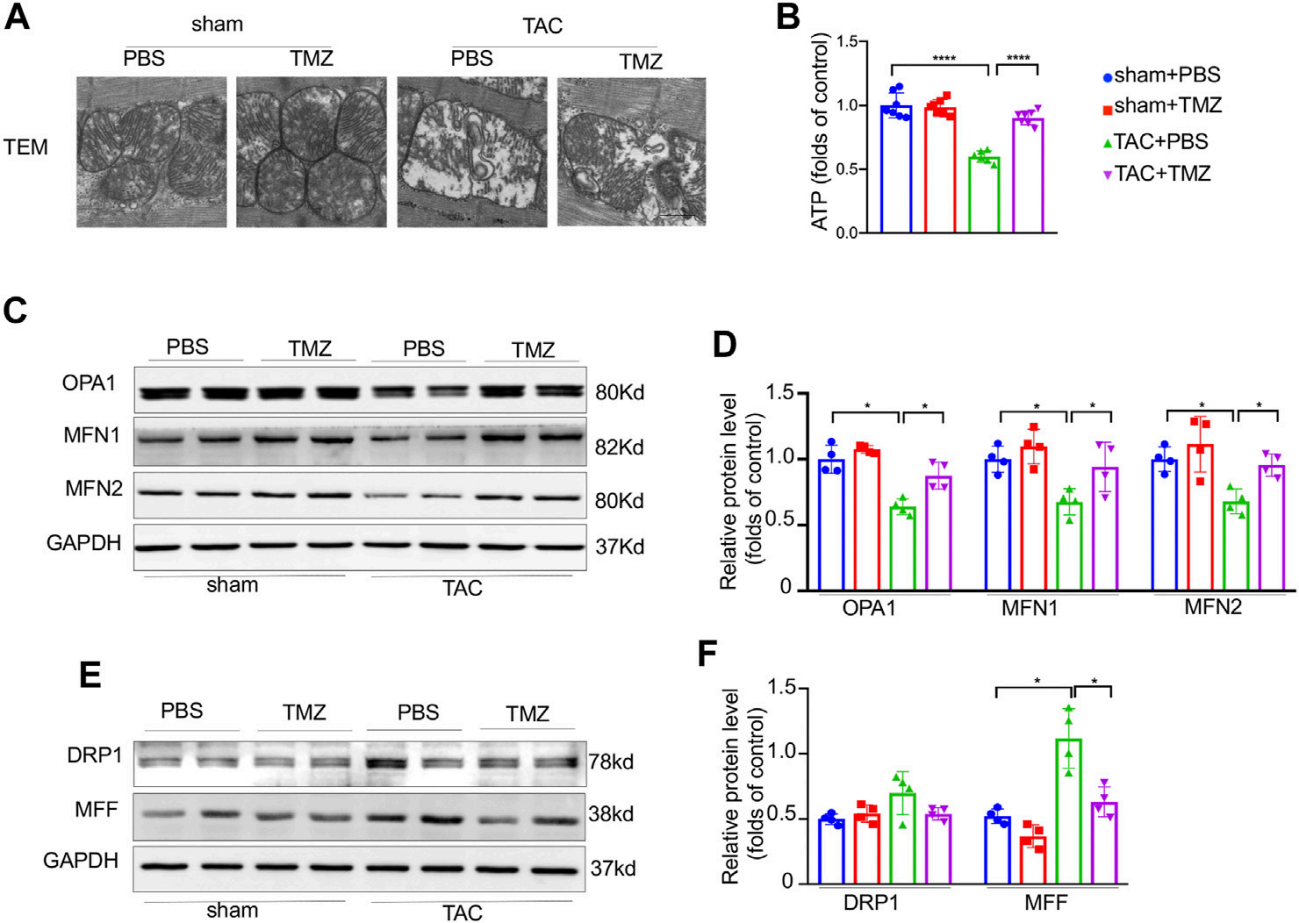
Trimetazidine improved the pressure overload-induced impairment of mitochondrial structure and function. **(A)** Representative ultrastructure image of mitochondria of mice heart (zoomed scale bar = 0.5 um). **(B)** Cardiac ATP content. **(C–F)** Representative western blots of mitochondrial dynamic protein and corresponding quantitative results. Four samples were included, Graphical data are shown as mean ± SEM. Two-way ANOVA was used for analysis. *p < 0.05, ****p < 0.0001 vs indicated group. TAC, Transverse aortic constriction; TMZ, Trimetazidine.

Since mitochondrial is a central modulator of energy production, mitochondria dysfunction and energy deprivation accelerate the deterioration of heart failure. We next evaluated whether trimetazidine attenuates pressure overload-induced mitochondrial dysfunction in the heart. Firstly, 4-weeks TAC induced mitochondrial swelling and crista deconstruction manifested with increased mitochondrial area and decreased mitochondrial volume density detected by TEM (Figure 2A). These alterations were sharply ameliorated by the treatment of trimetazidine. Secondly, a significant decrease of ATP content was observed in TAC mice, which was markedly blunted by the treatment of trimetazidine (ATP, TAC + PBS vs TAC + TMZ, *p* < 0.0001, Figure 2B). Thirdly, the representative fusion proteins, optic atrophy type 1 (OPA1), mitofusin 1 (Mfn1), mitofusin 2 (Mfn2) were significantly decreased after TAC, but reversed by trimetazidine (OPA1, TAC + PBS vs TAC + TMZ, *p* = 0.041. Mfn1, TAC + PBS vs TAC + TMZ, *p* = 0.018. Mfn2, TAC + PBS vs TAC + TMZ, *p* = 0.0103, Figure 2C,D). The protein level of fission protein dynamin-related protein 1 (Drp1) and mitochondrial fission factor (MFF) were elevated upon TAC, which were restrained by trimetazidine (MFF, TAC + PBS vs TAC + TMZ, *p* = 0.032, Figure 2E,F). Taken together, these findings suggested that trimetazidine improved pressure overload-induced impairment of mitochondrial structure and function.

### Trimetazidine Attenuated the Cardiac Insulin Resistance Evoked by Pressure Overload

**FIGURE 3:**
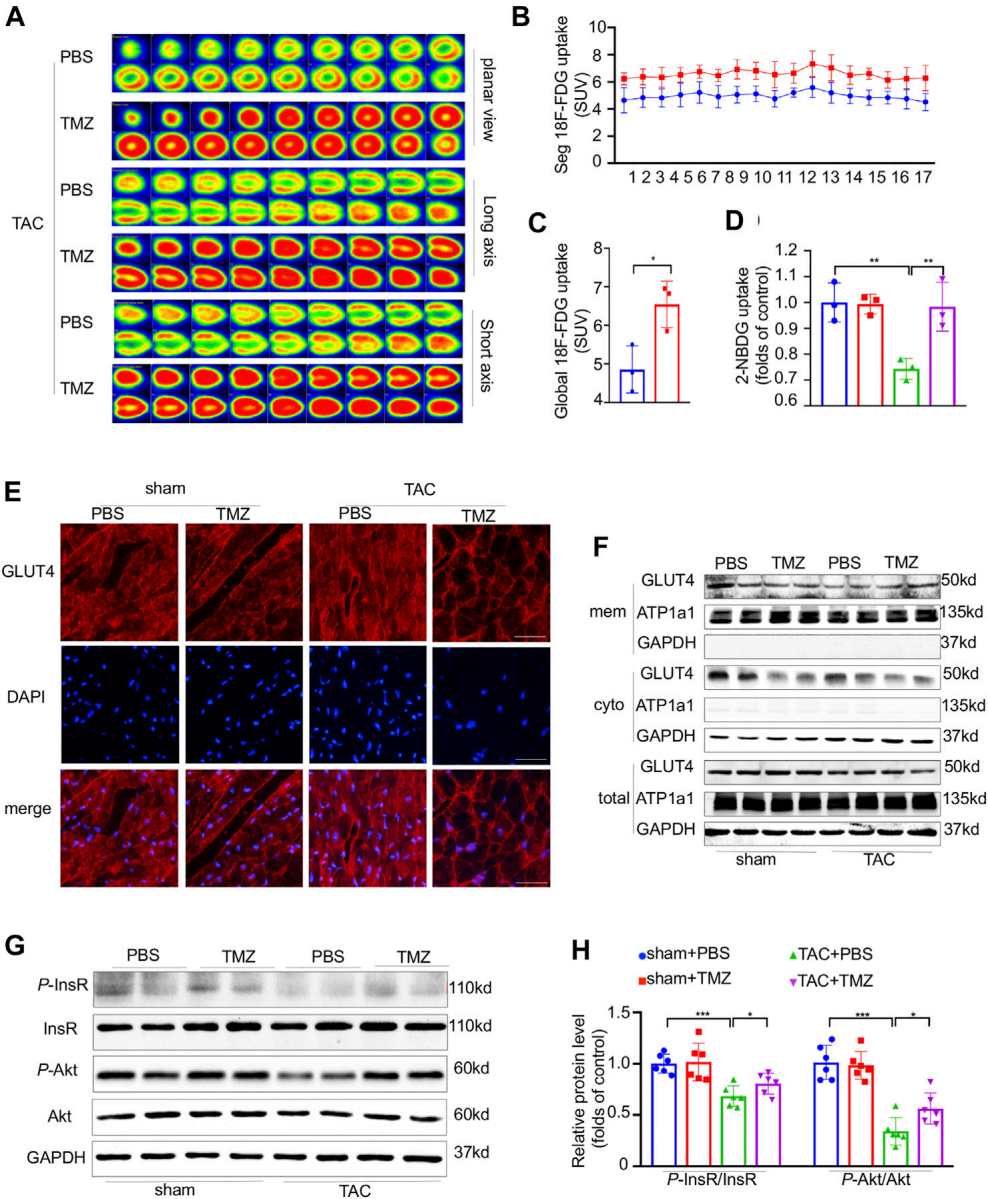
Trimetazidine attenuated the cardiac insulin resistance evoked by pressure overload. **(A)** Representative PET images showing the glucose uptake rate of mice heart, and corresponding calculated results **(B,C)** *n* = 3. **(D)** Glucose uptake rate of isolated adult cardiomyocytes by 2-NBDG assay, *n* = 3. **(E)** Immunofluorescence staining images labeling GLUT4 (red) and DAPI (blue) in mice heart tissue. Scale bar = 50 um. **(F)** Representative western blots of GLUT4 in membrane and cytoplasm in indicated groups, GAPDH was used as cytoplasm loading control and the total protein control, Na-K-ATPase was used as membrane loading control. **(G,H)** The protein levels of InsR, Akt and their phosphorylation states, *n* = 6. Graphical data are shown as mean ± SEM. Two-way ANOVA was used for analysis. *p < 0.05, **p < 0.01, ***p < 0.001 vs indicated group. TAC, Transverse aortic constriction; TMZ, Trimetazidine; SUV, Standard uptake value.

Trimetazidine is known as a specific inhibitor of β-oxidation, thus we firstly tested whether the cardioprotective effect brought by trimetazidine was based on the lipid regulation. However, no change was found in the serum and cardiac triglyceride (TG), total cholesterol (TC) level as well as the cell area of brown adipose tissue (BAT), white adipose tissue (WAT) and lipid metabolism related protein/gene levels (Supplementary Figure S1A–D, S4A–G). We next evaluated whether trimetazidine was involved in glucose metabolism, as shown in Supplementary Figure S1E–G, the AUC in TAC group was increased compared to sham group, indicating a glucose intolerance induced by TAC *in vivo* (sham + PBS vs TAC + PBS, 2 weeks, *p* < 0.01, 4 weeks, *p* < 0.01). However, the AUC was restrained in TAC + TMZ group, suggesting that TMZ played a beneficial role in glucose metabolism in pressure overload-induced heart failure (TAC + PBS vs TAC + TMZ, 2 weeks, *p* = 0.021, 4 weeks, *p* = 0.04). We further evaluated the glucose uptake in myocardium. Compared to the failing hearts of TAC mice treated by vehicle, the hearts from TAC mice treated by trimetazidine showed an increased glucose uptake as shown in Figure 3A–C (18F-FDG uptake, TAC + PBS vs TAC + TMZ, *p* = 0.034). The 2-NBDG assay testing on single isolated cardiomyocytes also showed a reduced glucose uptake after TAC, which was notably removed by the treatment of trimetazidine (2-NBDG uptake, TAC + PBS vs TAC + TMZ, *p* = 0.015, Figure 3D). As the most important insulin sensitive transporter subtype in myocardium, glucose transporter type 4 (GLUT4) translocated from the cytoplasm to membrane to uptake the glucose. We identified that the translocation of GLUT4 in LV tissue was significantly inhibited by TAC, which was reversed by the treatment of trimetazidine (TAC + PBS vs TAC + TMZ, *p* = 0.032, Figure 3E,F; Supplementary Figure S4). Collectively, these data indicated that although trimetazidine has no effect on the expression of GLUT4, it enhanced the membrane trafficking of GLUT4 in pressure overload states.

We next explored how trimetazidine increased the glucose uptake in heart. As shown in Figure 3G,H, pressure overload significantly reduced the phosphorylation of InsR (sham + PBS vs TAC + TMZ, *p* < 0.001), which were notably reversed by trimetazidine (TAC + PBS vs TAC + TMZ, *p* = 0.0163). The phosphorylation of Akt was reduced by pressure overload whereas preserved by trimetazidine (sham + PBS vs TAC + TMZ, *p* < 0.001. TAC + PBS vs TAC + TMZ, *p* = 0.0209) In all, the above data suggested trimetazidine elevated the glucose uptake and attenuated the myocardial insulin resistance evoked by pressure overload.

### Trimetazidine Inhibited the Pressure Overload-Evoked Myocardial Hypertrophy and Mitochondrial Dysfunction *In Vitro*

**FIGURE 4:**
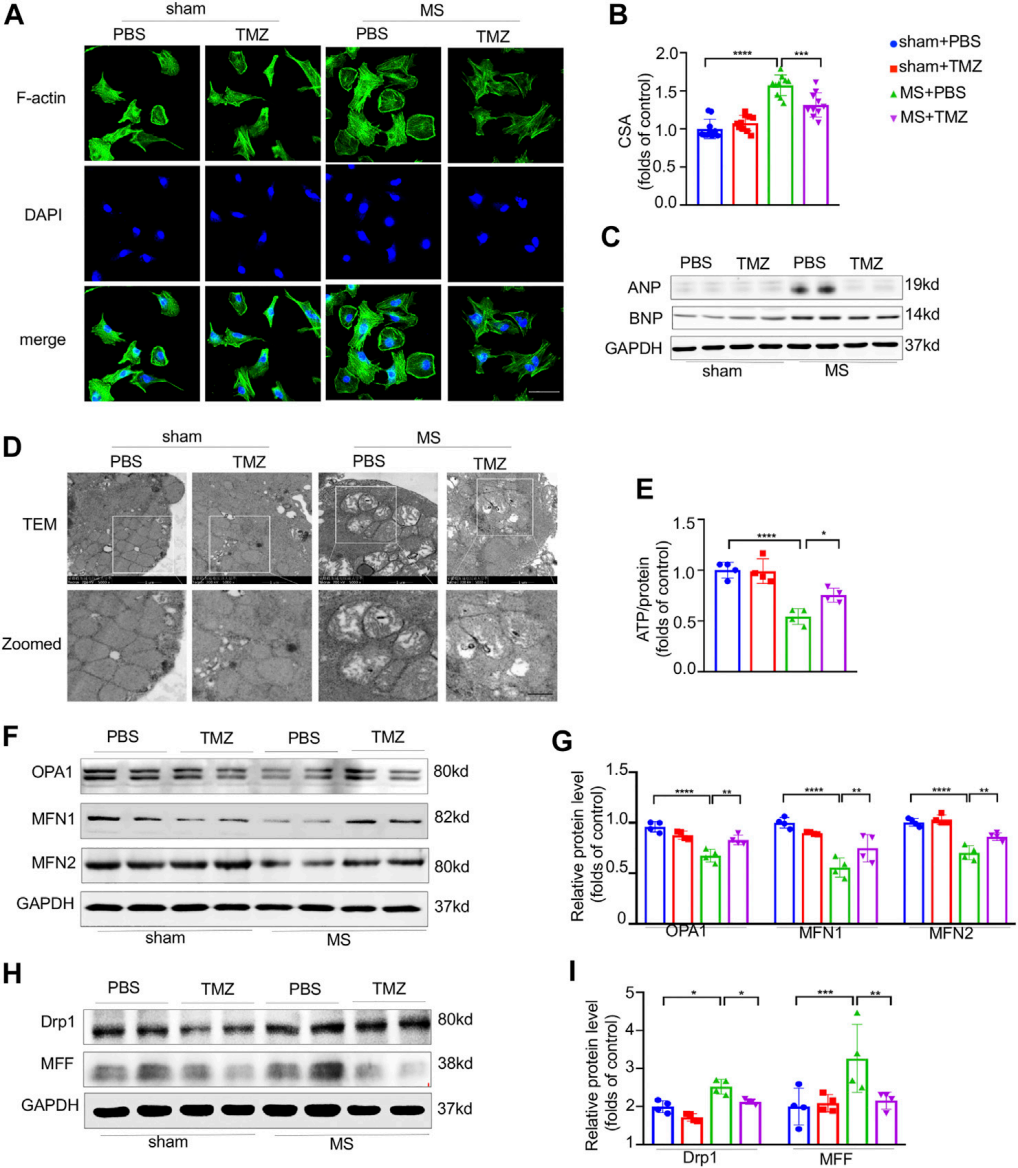
Trimetazidine inhibited the pressure overload-evoked myocardial hypertrophy and mitochondrial dysfunction *in vitro*. **(A)** Immunofluorescence staining of F-actin in NRCMs, nuclei were stained with DAPI, scale bar = 50 um. **(B)** NRCM area calculated from **(A)**, each group included 10 cells. **(C)** The protein levels of ANP and BNP in NRCM and their quantitative data collected from four independent samples. **(D)** Representative ultrastructure mitochondrial image of NRCMs. scale bar = 0.5 um. **(E)** The ATP content of NRCMs (*n* = 4). **(F,I)** Representative western blots of mitochondrial dynamic protein and corresponding quantitative results. Graphical data are shown as mean ± SEM. Two-way ANOVA was used for analysis. *p < 0.05, **p < 0.01, ***p < 0.001, ****p < 0.0001, vs indicated group. MS, Mechanical stretch; TMZ, Trimetazidine.

To verify the anti-hypertrophic effects of trimetazidine *in vitro*, NRCMs were mechanically stretched by using silicon plates. As shown in Figure 4A,B, mechanical stretch increased the CSA by 1.57-folds approximately (sham + PBS vs MS + PBS, *p* < 0.0001), which was notably abrogated by the treatment of trimetazidine (MS + PBS vs MS + TMZ, *p* < 0.001). In addition, we found a significant increased expression of hypertrophic associated proteins (ANP and BNP) in mechanically-stretched NRCMs, which was also reversed by trimetazidine (Figure 4C). We conducted an *in vitro* study to clarify whether trimetazidine had a direct protection on mitochondria by using TEM in cultured NRCMs (Figure 4D). We found numerous fragmented and disordered mitochondrial cristae in mechanically stretched NRCMs. Trimetazidine notably increased the mitochondrial cristae numbers and restored the cristae integrity and array. ATP content assay showed that stretched NRCMs exhibited a lower ATP content compared with untreated NRCMs (sham + PBS vs MS + PBS, *p* < 0.0001). The ATP level of stretched-MRCMs was approximately raised by 25% after the treatment of trimetazidine (MS + PBS vs MS + TMZ, *p* = 0.024, Figure 4E). Proteins associated with fusion process were decreased, whereas fission proteins such as Drp1 and MFF were increased under the stretch (Drp1, sham + PBS vs MS + PBS, *p* = 0.014. MFF, sham + PBS vs MS + PBS, *p* = 0.042). Trimetazidine moderated the changes of fusion and fission by inhibiting the downward trend of fusion proteins and the upward trend of fission proteins (Figure 4F–I, MS + PBS vs MS + TMZ, OPA1, *p* < 0.01, MFN1, *p* < 0.01, MFN2, *p* < 0.01, Drp1, *p* = 0.014, MFF, *p* < 0.01). These findings suggested that trimetazidine improved the mechanical stretch-induced mitochondrial dysfunction.

### Pressure Overload-Induced Impairment of Insulin Signaling Was Preserved by Trimetazidine *In Vitro*

**FIGURE 5:**
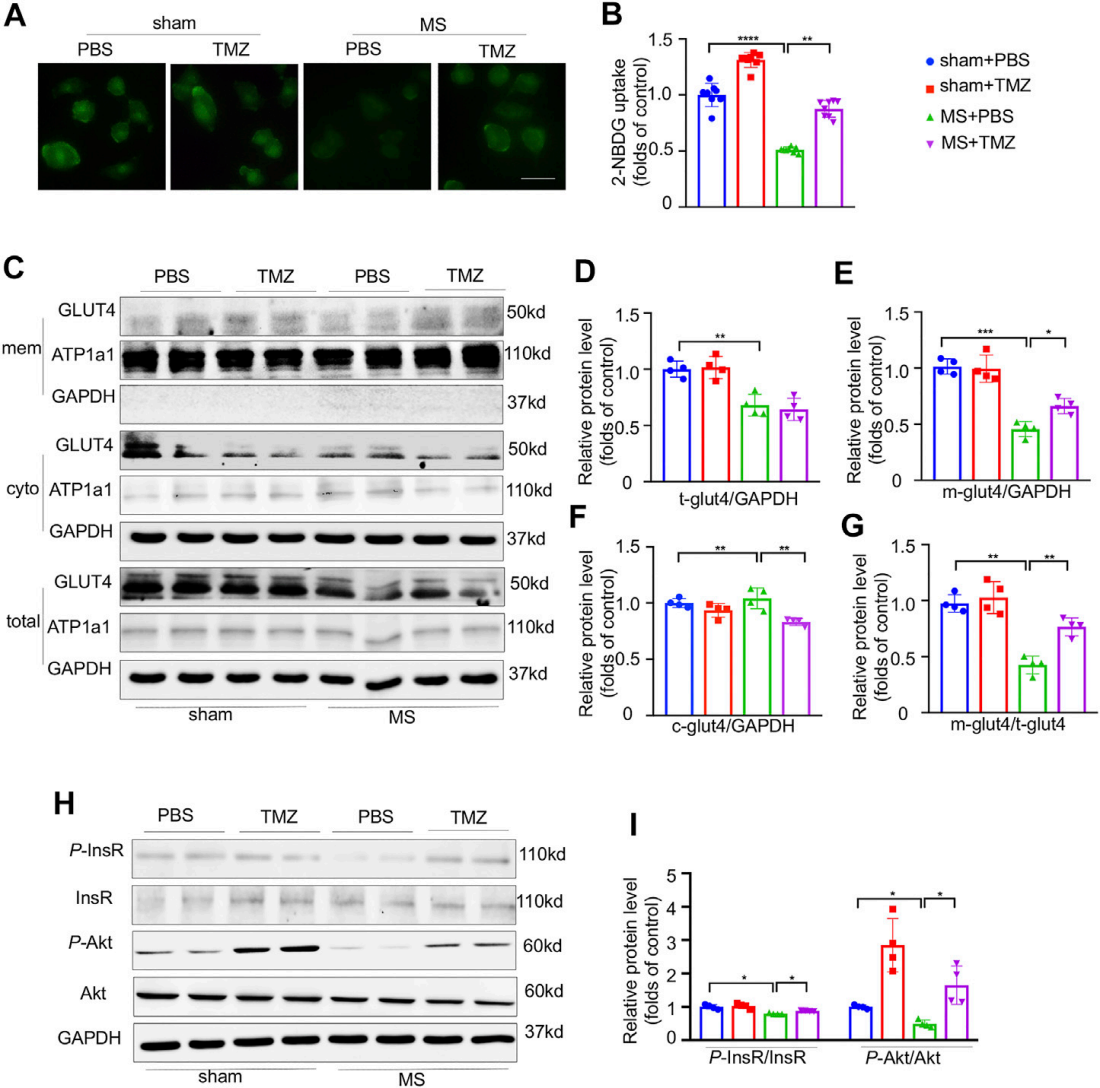
Pressure overload-induced impairment of insulin signaling was preserved by Trimetazidine *in vitro*. **(A,B)** The representative 2-NBDG fluorescence images and its quantitative analysis of H9C2 cell line. Scale bar = 100 um. **(C–G)** Representative GLUT4 western blots in NRCMs and correspondence quantitative analysis, Na-K-ATPase was used as the membrane loading control, and GAPDH for cytoplasm loading control. M-GLUT4, GLUT4 in the membrane. C-GLUT4, GLUT4 in the cytoplasm. T-GLUT4, total GLUT4. **(H,I)** The protein levels of InsR, Akt and their phosphorylation states. Graphical data are shown as mean ± SEM. Two-way ANOVA was used for analysis. *p < 0.05, **p < 0.01, ***p < 0.001, ****p < 0.0001, vs indicated group. MS, Mechanical stretch; TMZ, Trimetazidine.

Direct evidences from *in vitro* study indicated that mechanical stretch significantly reduced the glucose uptake as shown in Figure 5A. After mechanical stretch, treatment with trimetazidine remarkably increased the glucose uptake (shown as green staining) compared to vehicle-treated cardiomyocytes, indicating that trimetazidine improved the glucose uptake in cardiomyocytes under stretch (Figure 5B, sham + PBS vs MS + PBS, *p* < 0.0001, MS + PBS vs MS + TMZ, *p* < 0.01). Mechanical stretch reduced both the total and membrane GLUT4 content. Treatment with trimetazidine disproportionately increased the membrane amount of GLUT4 compared to vehicle-treated NRCMs under stretch, which was proved by a significantly higher ratio of membrane/cytoplasm GLUT4 (Figure 5C–G), implying that trimetazidine promoted the GLUT4 translocation from cytoplasm to membrane under mechanical stretch. Consistent with the *in vivo* study, a reduction in p-InsR were observed in mechanical-stretched NRCMs, but partially restored by the treatment of trimetazidine (Figure 5H,I, sham + PBS vs MS + PBS, *p* = 0.034, MS + PBS vs MS + TMZ, *p* = 0.0212). Meantime, p-Akt/Akt ratio was also decreased in mechanically-stretched NRCMs, which was suppressed by trimetazidine (sham + PBS vs MS + PBS, *p* = 0.041, MS + PBS vs MS + TMZ, *p* = 0.0309). These alterations implied that cardiac insulin signaling impairment provoked by pressure overload was relieved by trimetazidine.

### AMPK Was Essential for the Attenuation of Pressure Overload-Induced Mitochondria Dysfunction, Cardiac Insulin Resistance and Heart Failure by the Treatment of Trimetazidine

**FIGURE 6:**
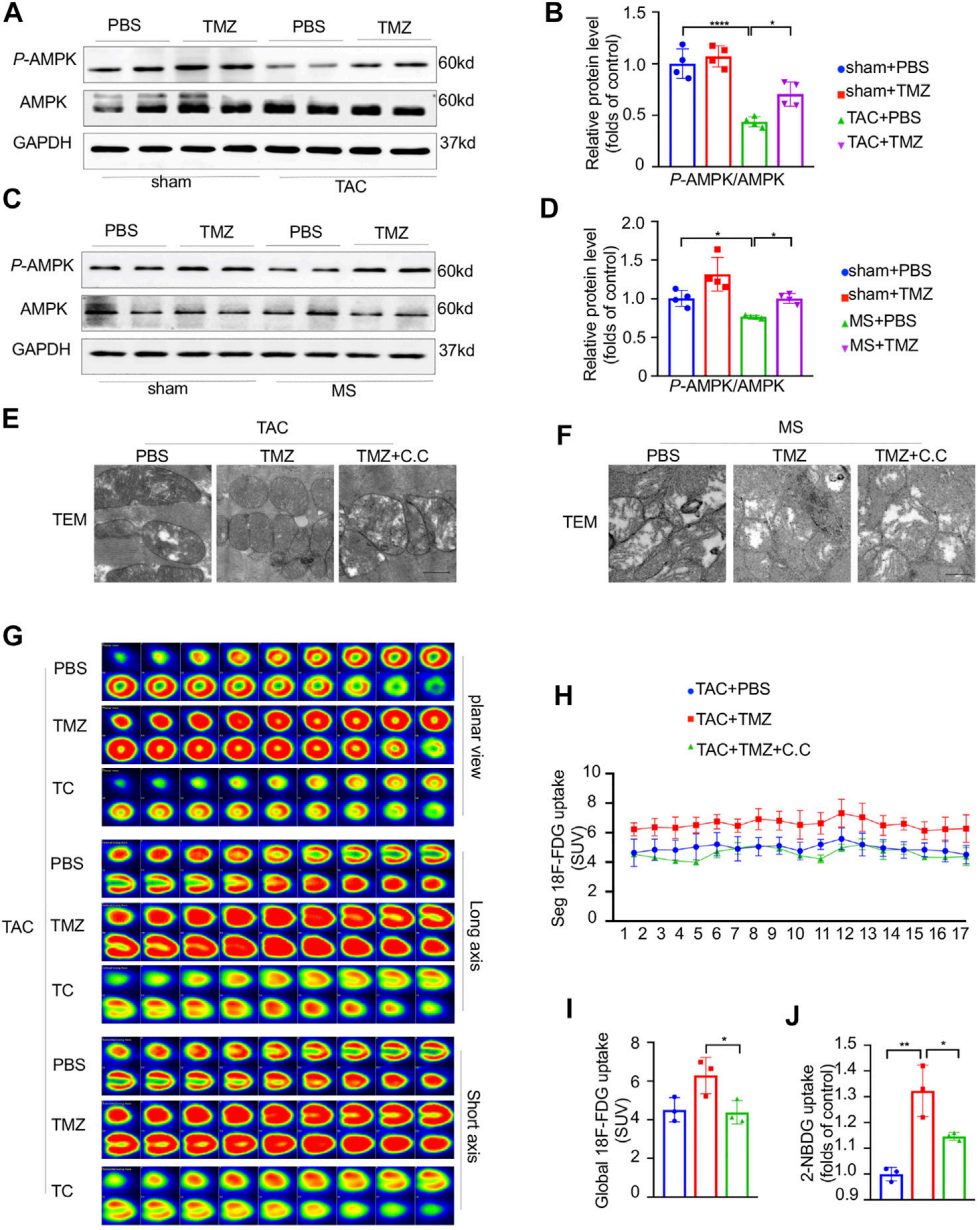
AMPK was essential for the attenuation of pressure overload-induced mitochondria dysfunction, cardiac insulin resistance and heart failure by the treatment of trimetazidine. **(A,B)** Representative western blots in mice heart and correspondence quantitative analysis. **(C,D)** Representative western blots in NRCMs and correspondence quantitative analysis. **(E,F)** Representative TEM of mice hearts and NRCM in indicated groups. Scale bar = 0.5 um. **(G)** The representative PET showing the cardiac glucose uptake and the quantitative analysis **(H,I)**, *n* = 3. **(J)** Glucose uptake rate of isolated adult cardiomyocytes by 2-NBDG uptake assay, *n* = 3. Graphical data are shown as mean ± SEM. Two-way ANOVA was used for analysis. *p < 0.05, **p < 0.01, ****p < 0.0001, vs indicated group. TAC, Transverse aortic constriction; MS, Mechanical stretch; C.C., Compound C; SUV, Standard uptake value.

To reveal the underlying molecular mechanism by which trimetazidine attenuates the myocardial glucose insensitivity and the resultant heart failure induced by pressure overload, we tested the key energy modulator, AMPK in the cardiomyocytes. As shown in Figure 6A–D, the phosphorylation of AMPKTyr172 was notably decreased by 54% (sham + PBS vs TAC + PBS, *p* < 0.0001) and 24% (sham + PBS vs MS + PBS, *p* = 0.0106) in response to pressure overload or mechanical stretch compared to control, respectively. The treatment of trimetazidine significantly increased the phosphorylation of AMPKTyr172 in pressure overloaded-cardiomyocytes compared to vehicle group (TAC + PBS vs TAC + MS, *p* = 0.018, MS + PBS vs MS + TMZ, *p* = 0.0201). These data intimated that AMPK was critically involved in the process of pressure overload-induced heart failure and might mediate the cardioprotective effects of trimetazidine.

Since the activation of AMPK is necessary for normal mitochondrial function, the TAC mice were pretreated by compound c (C.C), a specific AMPK inhibitor and then treated with trimetazidine or vehicle. Firstly, we evaluated whether the activation of AMPK plays a critical role in the trimetazidine-induced cardioprotection against pressure overload. The reduced EF in TAC mice were ameliorated by trimetazidine. However, these beneficial effects led by trimetazidine were largely abrogated by the pretreatment of C.C (TAC + TMZ vs TAC + TMZ + C.C, *p* = 0.02, Supplementary Figure S2B, Supplementary Table S4). Additionally, the increased CSA and cardiac fibrosis in TAC mice were also restrained by trimetazidine, whereas disappeared when the TAC mice were pretreated with C.C (TAC + TMZ vs TAC + TMZ + C.C, CSA, *p* < 0.001, PVF, *p* < 0.01, MFA, *p* < 0.01, Supplementary Figure S2A,C–E). These findings illustrated that inhibiting the activation of AMPK partially countermand the cardioprotective effects of trimetazidine. Secondly, we observed a notable destroy of mitochondrial ultrastructure, as shown in Figure 6E, which was preserved by trimetazidine significantly. However, pretreatment with C.C abrogated the protective effect of trimetazidine against pressure overload, indicating that trimetazidine-associated mitochondrial protection is mediated by AMPK. Thirdly, trimetazidine restored the unbalance of mitochondrial fusion and fission proteins caused by pressure overload and mechanical stretch, which was also abrogated by the inhibition of AMPK (Supplementary Figure S2F).

As shown in Figure 6G–I, the improvement of cardiac glucose uptake led by trimetazidine in the heart was totally abolished by the pretreatment of C.C (TAC + TMZ vs TAC + TMZ + C.C, *p* = 0.0119). 2-NBDG uptake assay unraveled that the glucose uptake rate of single cardiomyocyte was elevated by trimetazidine in the cardiomyocytes of TAC mice, but removed by C.C (MS + TMZ vs MS + TMZ + C.C, *p* = 0.0302, Figure 6J). The increased distribution of GLUT4 led by trimetazidine was inhibited by C.C (Supplementary Figure S2G), indicating that AMPK was responsible for the GLUT4 translocation to the cell membrane by trimetazidine. Moreover, the improvement of p-Akt/Akt and p-InsR/InsR were also abrogated by C.C (MS + TMZ vs MS + TMZ + C.C, P-Akt/Akt, *p* = 0.0218, P-InsR/InsR, *p* = 0.017, Supplementary Figure S2H,I). These findings implied that AMPK was critically involved in the protective effects of trimetazidine on cardiac insulin resistance.

## Discussion

**FIGURE 7:**
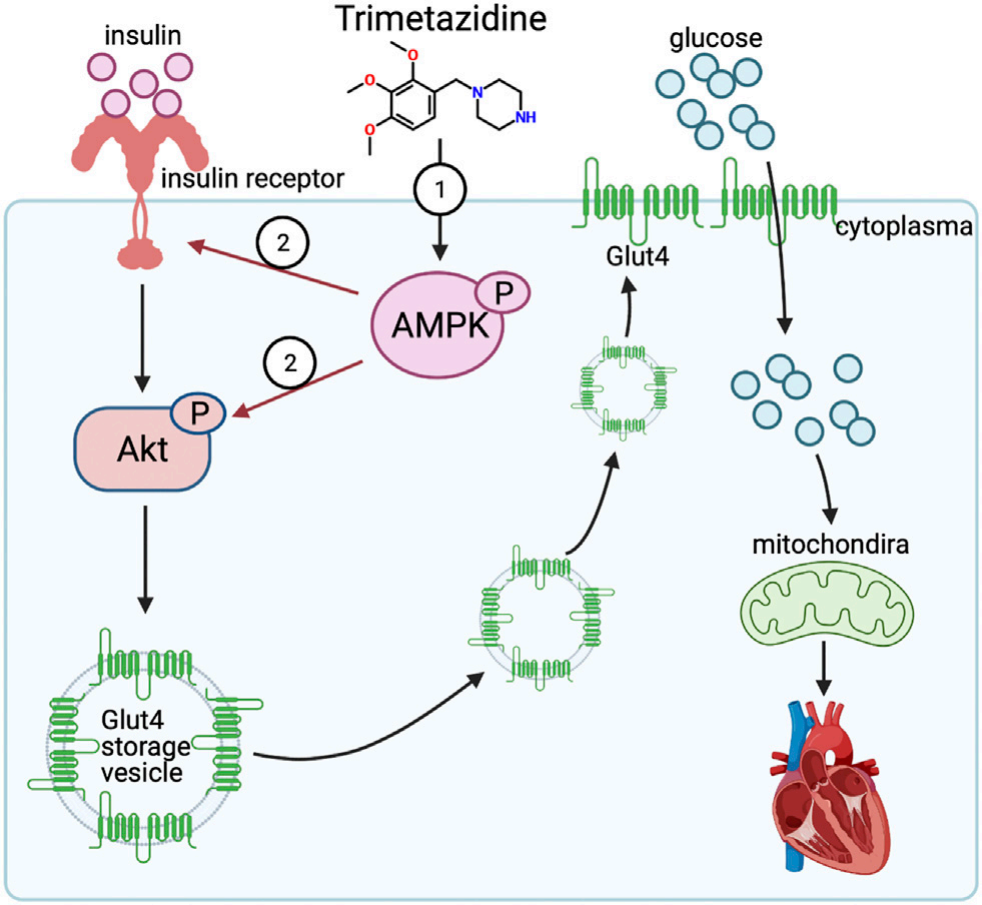
Trimetazidine activates insulin signaling pathway evident by enhanced expression of insulin receptor and activated Akt via AMPK, thus elevating the glucose uptake rate and improving the mitochondrial function against pressure overload induced heart failure.

In the present study, we showed a novel cardioprotective effect of trimetazidine against the pressure overload-induced CHF through enhancing the activation of AMPK, by which trimetazidine improved the insulin sensitivity and resultantly relieved mitochondrial dysfunction (Figure 7). In particular, our results showed for the first time that trimetazidine has a potentially attractive indication for pharmacological treatment of hypertensive congestive heart failure. There are four key findings in the present study. First, trimetazidine notably relived the CHF induced by pressure overload. Second, trimetazidine protected the mitochondria against pressure overload and increased the ATP supply in cardiomyocytes. Third, trimetazidine ameliorated the impairment of insulin signaling pathway and increased the cardiac glucose uptake in the pressure overload mouse model. Four, we identified AMPK as a molecular target of trimetazidine. Therefore, we here provide a new perspective of trimetazidine in the treatment of CHF.

Trimetazidine was a cytoprotective anti-anginal agent and used in the treatment of ischemic heart disease in the last 40 years. Trimetazidine improved myocardial glucose utilization through inhibition of long-chain 3-ketoacyl CoA thiolase activity, which results in a reduction in fatty acid oxidation and a stimulation of glucose oxidation in ischemic heart disease. High fatty acid oxidation rates are detrimental during ischemia due to an inhibition of glucose oxidation leading to uncoupling of glycolysis and an increase in protein production, which has the potential to accelerate sodium and calcium overload in the heart, leading to an exacerbation of ischemic injury and decreased cardiac efficiency during reperfusion (Lopatin et al., 2016). We here explored whether trimetazidine had any effect on the fatty acid and lipid metabolism in mice. However, neither serum and myocardium TC and TG levels, nor the accumulation of lipid droplets in liver, the enlargement of white adipose tissue and brown adipose tissue, the alterations of cardiac lipid metabolism gene/protein levels were found in TAC mice.

Our data showed that heart failure manifested by decreased EF and enlarged heart chamber were ameliorated with the treatment of trimetazidine, which was consistent with the clinical findings (McCarthy et al., 2016). We also observed that cardiac fibrosis were relieved by trimetazidine in agreement with previous report (Zhang et al., 2016). However, how trimetazidine functioned as a cardioprotective agent in heart failure remains unknown.

Accumulating evidences had shown that heart failure is always accompanied by an inadequate energy supply of cardiomyocytes. Targeting the metabolic disorder of the cardiomyocytes had been raised as a promising therapeutic target for the heart failure. Since trimetazidine was beneficial for the myocardial glucose utilization under myocardial ischemia, we hypothesized that trimetazidine may improve the abnormal myocardial metabolism in the pressure overload-induced heart failure.

Mitochondrion is known as the “power house” of the cell which produces nearly 90% energy when oxygen is available (Worldwide trends in blood pressure from 1975 to 2015: a pooled analysis of 1,479 population-based measurement studies with 19·1 million participants., 2017). Accumulating evidences showed that disturbance affecting mitochondrial fusion and fission process may affect its homeostasis, thus deteriorated cardiac function (Hall et al., 2014; Knowlton et al., 2014; Song et al., 2017). Early postnatal cardiomyocyte-specific Drp1 deficiency was lethal, which highlighted the important role of fission process in the development (Song et al., 2015). Mitochondrial fusion process was also vital for survival evidenced by eccentric heart formed in Mfn1/Mfn2 deficiency mice (Song et al., 2017). Besides, phosphorylated Mfn1 caused by extracellular regulated kinase (ERK) stimulated mitochondrial apoptosis (Cook et al., 2017). Therefore, both fusion protein and the fission protein were critical for mitochondrial function. We revealed that trimetazidine preserved the mitochondrial substructure. In line with the structural alterations, the generation of ATP was also elevated in the presence of trimetazidine, which was consistent with previous report that trimetazidine exerted its protective role on muscle atrophy via improving mitochondrial biogenesis (Molinari et al., 2017). For many years, researchers believed that the mitochondrion constantly changes its shape to adjust to the different energy demand (Ausman et al., 2018). While recent evidences showed that mitochondrial morphology is largely determined by the homeostasis of fusion and fission process (Youle and van der Bliek, 2012; Dorn, 2016). Expectedly, the unbalance of fusion and fission in response to pressure overload was observed both *in vivo* and *in vitro*. The fusion-associated proteins MfN1/2 and OPA1 were dramatically decreased whereas fission-related proteins Drp1 and MFF were oppositely increased. Trimetazidine was shown to keep the balance with elevation of Mfn1/2 and OPA1.

Accumulating evidences had shown that insulin resistance led to mitochondrial dysfunction (Gonzalez-Franquesa and Patti, 2017). Clinical observations had shown that mitochondrial oxidative capacity was decreased in obese and T2DM individuals. Moreover, microarray studies have successively strengthened this association by providing evidence that genes involved in oxidative metabolism and under the control of PGC1α are down-regulated in the individuals with a family history of T2DM and patients affected by T2DM compared to healthy controls (Mootha et al., 2003). Later, no changes were observed in the protein levels of PGC1α, complex I, II and V of the ETC in those with insulin resistance in a study which induced a transitory insulin resistance by overfeeding the sedentary non-obese individuals, showing that insulin resistance occurred ahead of mitochondrial dysfunction (Sergi et al., 2019). Furthermore, genetic deletion of IRS1/2 in mice exhibiting liver mitochondrial dysfunction (Franko et al., 2017), which underlined the direct association between insulin resistance and mitochondrial dysfunction. Further study revealed that insulin resistance suppressed the transcription factor foxhead box O1(FOXO1), whose downstream protein directly disrupted the mitochondrial ETC complexes (Cheng et al., 2009). Thus, the amelioration of insulin resistance is expected to improve mitochondrial function.

Hence, we presumed that the mechanism by which trimetazidine protected the mitochondrial function and structure is dependent of its promotion of cardiac insulin sensitivity under the pressure overload. The effect of trimetazidine on the heart was accessed by the PET and 2-NBDG assay in the present study. PET was one of the most effective methods to evaluate the glucose uptake rate in different tissue and organ (Rijzewijk et al., 2009), thus adopting PET to show the myocardium glucose uptake offered a straightforward impression and a precise evaluation on glucose uptake. Moreover, the glucose uptake rate of the single cardiomyocytes was also accessed *in vitro*, which not only supported the trimetazidine effects in the aspect of myocardium glucose uptake, but also underlined the cardiomyocytes contribution to the whole beneficial effects. Trimetazidine promoted the glucose oxidation by inhibiting the β-oxidation. In particular, Acetyl CoA derived from fatty acid oxidation inhibited the rate-limiting enzyme of glucose oxidation, pyruvate dehydrogenase (PDH). Thus lowering β-oxidation conversely enhanced the glucose oxidation (Lopatin et al., 2016). Our study confirmed that trimetazidine acted on glucose uptake which was the upstream of glucose oxidation, which perfected the understanding of trimetazidine on glucose metabolism. Both *in vivo* and *in vitro* study demonstrated that trimetazidine enhanced the glucose uptake and promoted the glucose utilization of the heart under TAC. Trimetazidine applied in idiopathic dilated cardiomyopathy patients, of which trimetazidine improved whole body insulin sensitivity and glucose control in these insulin resistant patients (Tuunanen et al., 2008). According to our observations, trimetazidine enhanced the glucose uptake, improved the insulin resistance, and resultantly rescued the impaired substrates supply for mitochondria, therefore improved the heart failure induced by pressure overload.

Insulin signaling pathway in the cardiomyocytes plays an essential role in the regulation of glucose uptake and utilization (Haeusler et al., 2018). Once insulin binds to insulin receptor in the myocardial membrane, it induces a conformational change of InsR (Weis et al., 2018), then triggers its auto-phosphorylation, which in turn increases its tyrosine kinase activity for other substrates, such as IRS1/2 (Petersen and Shulman, 2018). In the cascade of insulin signaling, Akt serves as a key molecular in mediating diversity cellular processes. Selectively phosphorylating Akt at ser473 was sufficient to stimulate GLUT4 translocation (Ng et al., 2008). We found that p-InsR/InsR and the p-Akt/Akt ratio were reduced after 4 weeks of TAC, indicating an impaired insulin-signaling pathway in response to pressure overload.

AMPK is a highly conserved master regulator of metabolism that functions as an energy sensor (Day et al., 2017). The deactivation of AMPK led to cardiac insulin resistance via a direct inhibition of AS160 and Akt. Activating AMPK improved insulin resistance in skeletal muscle through facilitating GLUT4 trafficking to the cellular membrane via phosphorylating TBC1 domain family member 1 (TBC1D1) (Hardie, 2013; Kjøbsted et al., 2019). AMPK also promoted the GLUT4 expression by a positive loop with peroxisome proliferator-activated receptor β (PPARβ) (Koh et al., 2019). Thus, AMPK was a promising target in augmenting insulin sensitivity. Our preliminary data showed that trimetazidine activated AMPK in a dose-dependent manner. AMPK and Akt are the two main modulators in energy metabolism (Zhao et al., 2017). Phosphorylated AMPK activated Akt through phosphorylating IRS1 at Ser794 (Tzatsos and Tsichlis, 2007). Besides, inhibition of AMPK blocked the irisin-mediated increase in phosphorylation of eNOS and Akt in endothelial cells and vasodilation in mesenteric arteries (Fu et al., 2016). In our study, the p-Akt/Akt was elevated in the presence of trimetazidine. Taken the relationship of AMPK and Akt into consideration, it was logically to think that trimetazidine activated AMPK followed by the activation of Akt.

In summary, the present study not only provided a possibility that trimetazidine might be used in hypertensive heart failure patients, but also connected glucose uptake with trimetazidine directly. Our results further suggested a molecular-based link between the development of the heart failure with the myocardial metabolic disorder and illustrated that trimetazidine protected mitochondrial function and improved myocardial metabolism by activating AMPK.

## Data Availability

The raw data supporting the conclusions of this article will be made available by the authors, without undue reservation.

## Supplementary Material

Supplementary Figure S1 Trimetazidine altered glucose metabolism instead of lipid metabolism in vivo. **(A,B)** The serum TC and TG levels after TMZ treatment in 0, 2, 4 weeks. TC, triglyceride; TG, cholesterol. **(C)** The representative H&E staining of liver, WAT, BAT section and their correspondence across sectional area **(D)** WAT, White Adipose Tissue; BAT, Brown Adipose Tissue. scale bar = 50 um. **(E)** The area under the curve (AUC) of blood glucose in 2 weeks of post TAC and 4 weeks of post TAC. **(F,G)** The blood glucose of 2 weeks of post TAC and 4 weeks of post TAC. **p* < 0.05, ***p* < 0.01, *****p* < 0.0001, vs indicated groups. TAC, Transverse aortic constriction; TMZ, Trimetazidine; C.C., Compound C.

Supplementary Figure S2 AMPK was essential for the attenuation of pressure overload-induced mitochondria dysfunction, cardiac insulin resistance and heart failure by the treatment of trimetazidine. **(A)** Representative histological images of H&E (scale bar = 100 um) and WGA staining (scale bar = 100 um), representative cardiac section stained with Masson’s trichrome (scale bar = 100 um). **(B)** Left ventricular ejection fraction (EF). **(C)** Relative across sectional area of cardiomyocytes calculated from WGA staining. **(D,E)** Quantitative analysis of PVF and MFA. PVF, Perivascular Fibrosis Area; MFA, Myocardial Fibrosis Area. **(F)** Representative western blots of mitochondrial dynamic protein in mice heart. **(G)** The immunofluorescence staining of GLUT4 (red) and DAPI (blue) in heart tissue, scale bar = 50 um. **(H,I)** The protein levels of InsR, Akt and their phosphorylation states. Data generated from four replicates. **p* < 0.05, ***p* < 0.01, ****p* < 0.001, *****p* < 0.0001, vs indicated groups. TAC, Transverse aortic constriction; TMZ, Trimetazidine; C.C., Compound C; TC, TMZ+C.C.

Supplementary Figure S3 Quantitative analysis of Figure 3F data generated from four replicates. **p* < 0.05, ***p* < 0.01, vs indicated groups. TAC, Transverse aortic constriction; TMZ, Trimetazidine.

Supplementary Figure S4 Trimetazidine didn’t alter cardiac lipid metabolism in vivo. **(A,B)** The cardiac TC and TG levels after TMZ treatment in 4 weeks. TC, triglyceride. TG, cholesterol. **(C)** The relative mRNA levels of cardiac lipid metabolism gene levels, including CD36, PPARα (peroxisome proliferator-activated receptor-alpha), FASN (fatty acid synthase), CPT1a (carnitine palmitoyl-transferase-1a), CPT1b (carnitine palmitoyl-transferase-1b), ACC1 (acetyl CoA carboxylase 1) in myocardium after TMZ treatment in 4 weeks. **(D)** Representative western blots of cardiac lipid metabolism protein and corresponding quantitative results. ***p* < 0.01, vs indicated groups. TAC, Transverse aortic constriction; TMZ, Trimetazidine.

Supplementary Figure S5 Trimetazidine didn’t alter the protein expression of Glut1. **(A,B)** Representative western blots of Glut1 and corresponding quantitative results. Glut1, type I glucose transporter. ***p* < 0.01, vs indicated groups. TAC, Transverse aortic constriction; TMZ, Trimetazidine.

Supplementary Table S1 Primer sequences for quantitative real-time PCR.

Supplementary Table S2 Key resource table.

Supplementary Table S3 Echocardiographic and hemodynamics data of C57BL/6 mice at 4 weeks after sham or TAC surgery. HR, heart rate; BPM, beat per minute; LVAW;d, LV anterior wall thickness at diastole; LVID;d, LV internal diameter at diastole; LVPW;d, LV posterior wall thickness at diastole; LVAW;s, LV anterior wall thickness at systole; LVID;s, LV internal diameter at systole;LVPW;s, LV posterior wall thickness at systole; FS, fractional shortening; EF, ejection fraction. Data shown as mean ± SEM.**p* < 0.05, vs Sham+PBS; #*p* < 0.05, vs TAC+PBS.

Supplementary Table S4 Echocardiographic and hemodynamics data of C57BL/6 mice at 4 weeks after TAC surgery in the presence of Compound C.C. HR, heart rate; BPM, beat per minute; LVAW;d, LV anterior wall thickness at diastole; LVID;d, LV internal diameter at diastole; LVPW;d, LV posterior wall thickness at diastole; LVAW;s, LV anterior wall thickness at systole; LVID;s, LV internal diameter at systole;LVPW;s, LV posterior wall thickness at systole; FS, fractional shortening; EF, ejection fraction. Data shown as mean ± SEM. **p* < 0.05, vs Sham+PBS; #*p* < 0.05, vs TAC+TMZ.

## Abbreviations

2-NBDG, 2-Deoxy-2- [(7-nitro-2,1,3-benzoxadiazol-4-yl) amino]-d-glucose; Akt, protein kinase B; AMPK, AMP-activated protein kinase; ANOVA, Analysis of Variance. ANP, atrial natriuretic peptide; ATP, adenosine triphosphate; BAT, brown adipose tissue; BNP, brain natriuretic peptide; C.C, compound C; CHF, chronic heart failure; CSA, cross-sectional area; Drp1, dynamin-related protein 1; ERK, extracellular regulated kinase; ETC, electron transfer chain; FBS, fetal bovine serum; FDG, flurodeoxyglucose; FFA, free fatty acid; FOXO1, transcription factor foxhead box O1; GLUT4, glucose transporter type IV; GSV, glucose storage vesicle; InsR, insulin receptor; IRS1, insulin receptor substrate 1; LV, left ventricular; MFF, mitochondrial fission factor; MFN1, mitofusin 1; MFN2, mitofusin 2; NRCM, neonatal rat cardiomyocyte; OPA1, optic atrophy type 1; PDH, pyruvate dehydrogenase; PET, positron emission tomography; PGC1α, peroxisome proliferator-activated receptorγcoactivator-1α; PPARβ, peroxisome proliferator-activated receptor β; ROI, region of interest; SUV, standardized uptake value; T2DM, type 2 diabetes mellitus; TAC, transverse aortic constriction; TBC1D1:TBC1 domain family member 1; TC, total cholesterol; TEM, Transmission Electron Microscope; TG, triglyceride; WAT, white adipose tissue; WGA, wheat germ agglutinin.
