# Supplementary figures and images for: Trimetazidine Attenuates Heart Failure by Improving Myocardial Metabolism via AMPK
Source: Front Pharmacol. 2021 Sep 15;12:707399. doi: 10.3389/fphar.2021.707399 (PMC8479198; doi:10.3389/fphar.2021.707399)

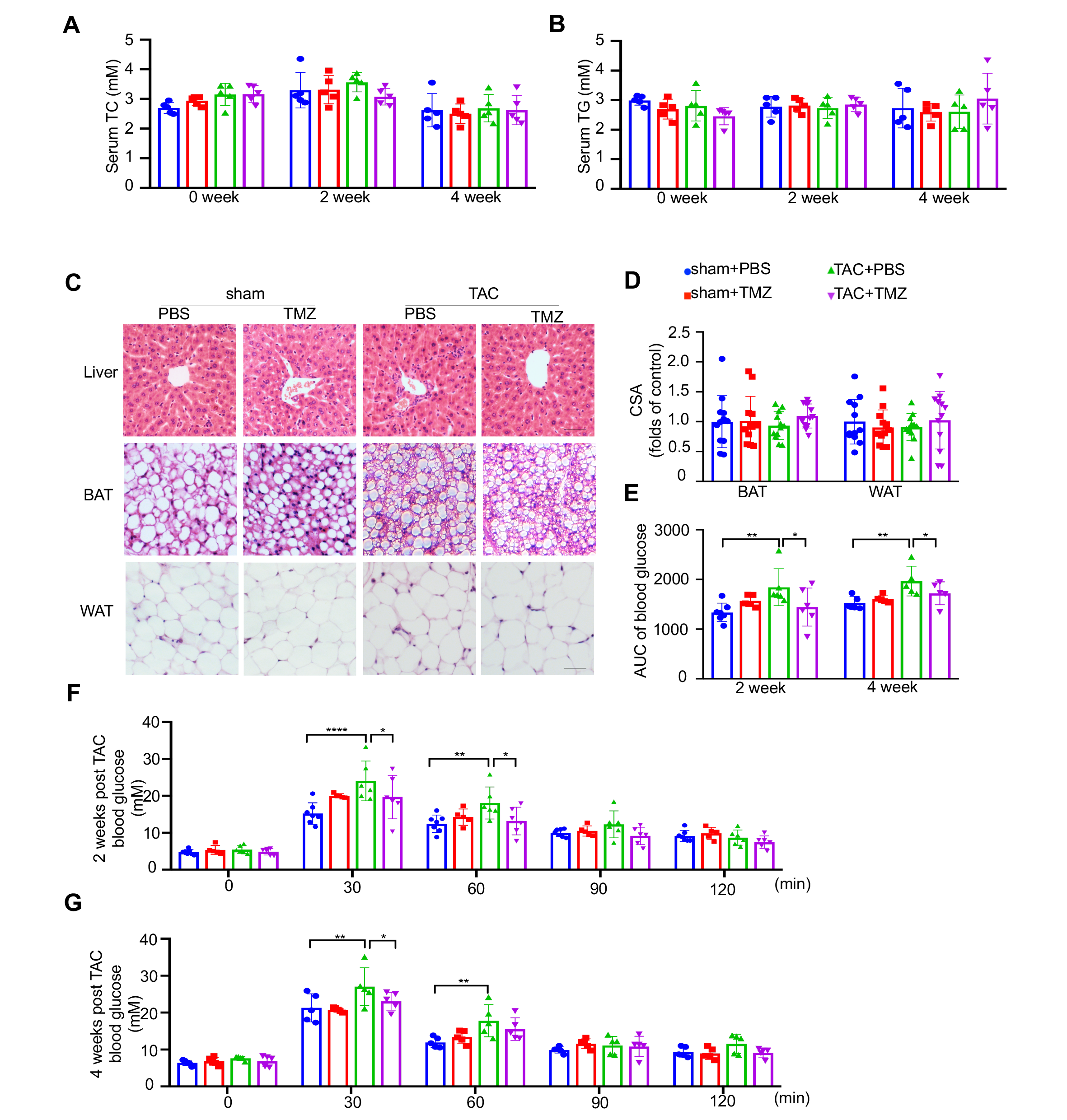

Supplement: Supplementary file 1 [file Image1.TIF]

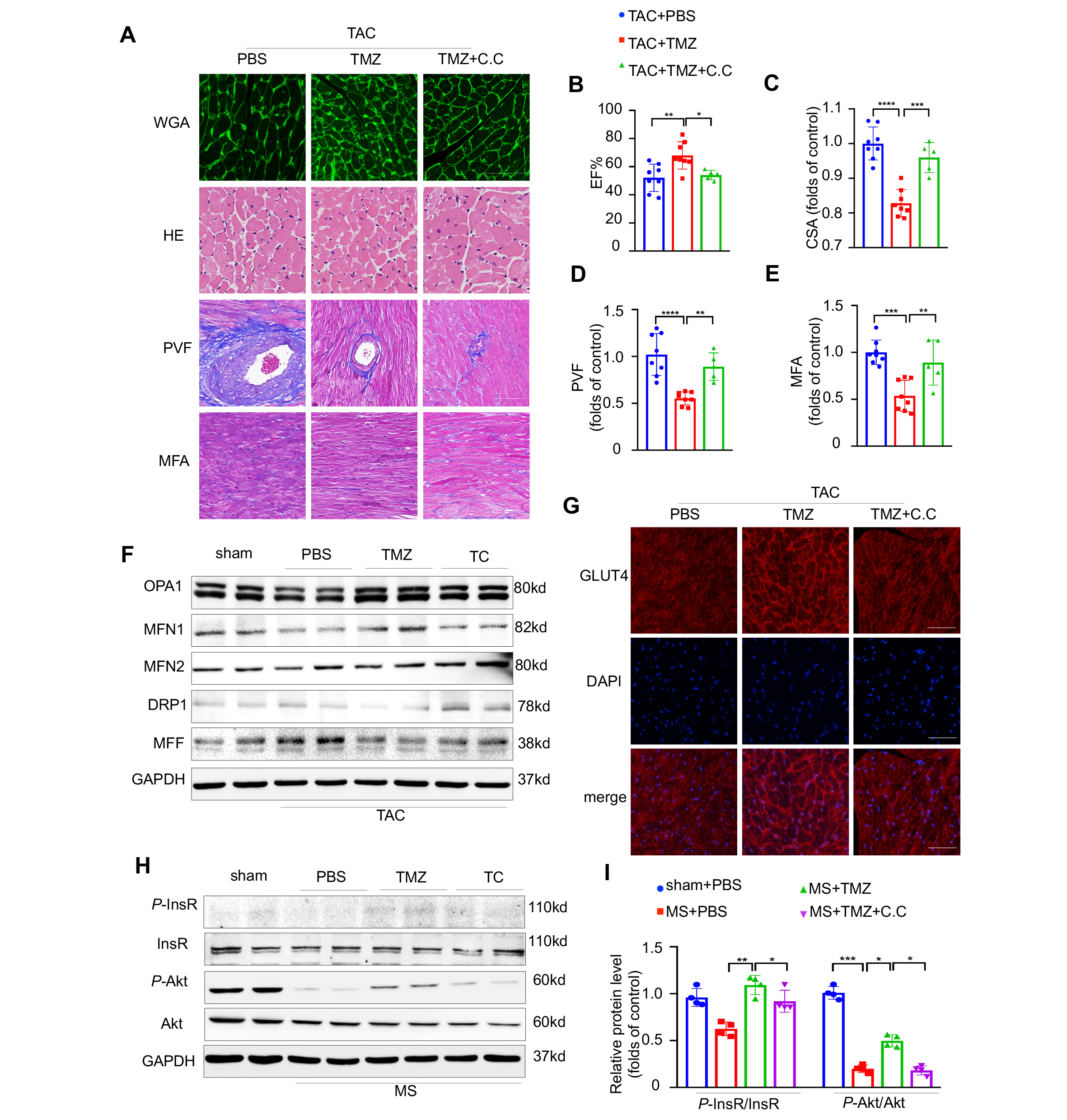

Supplement: Supplementary file 2 [file Image2.JPEG]

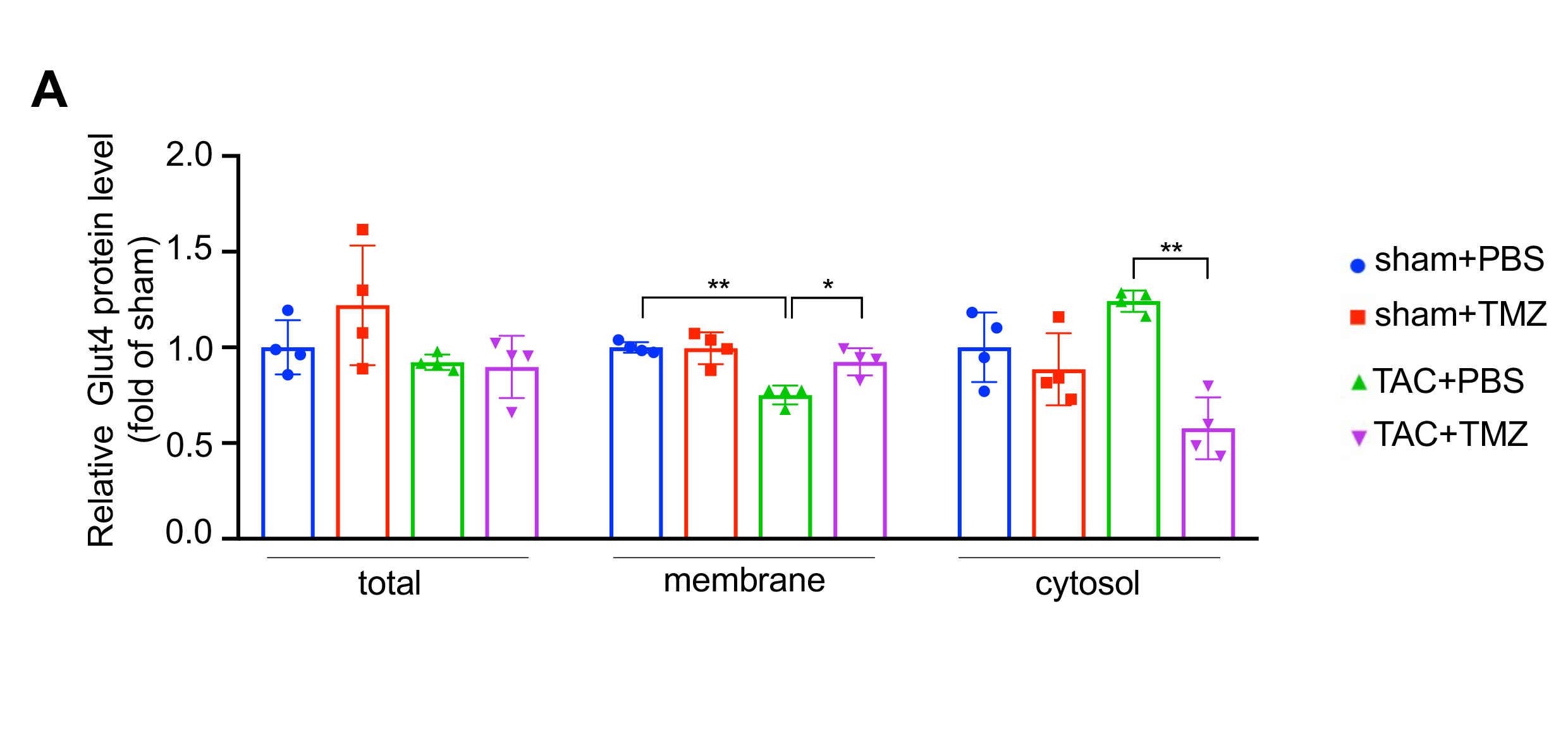

Supplement: Supplementary file 3 [file Image3.JPEG]

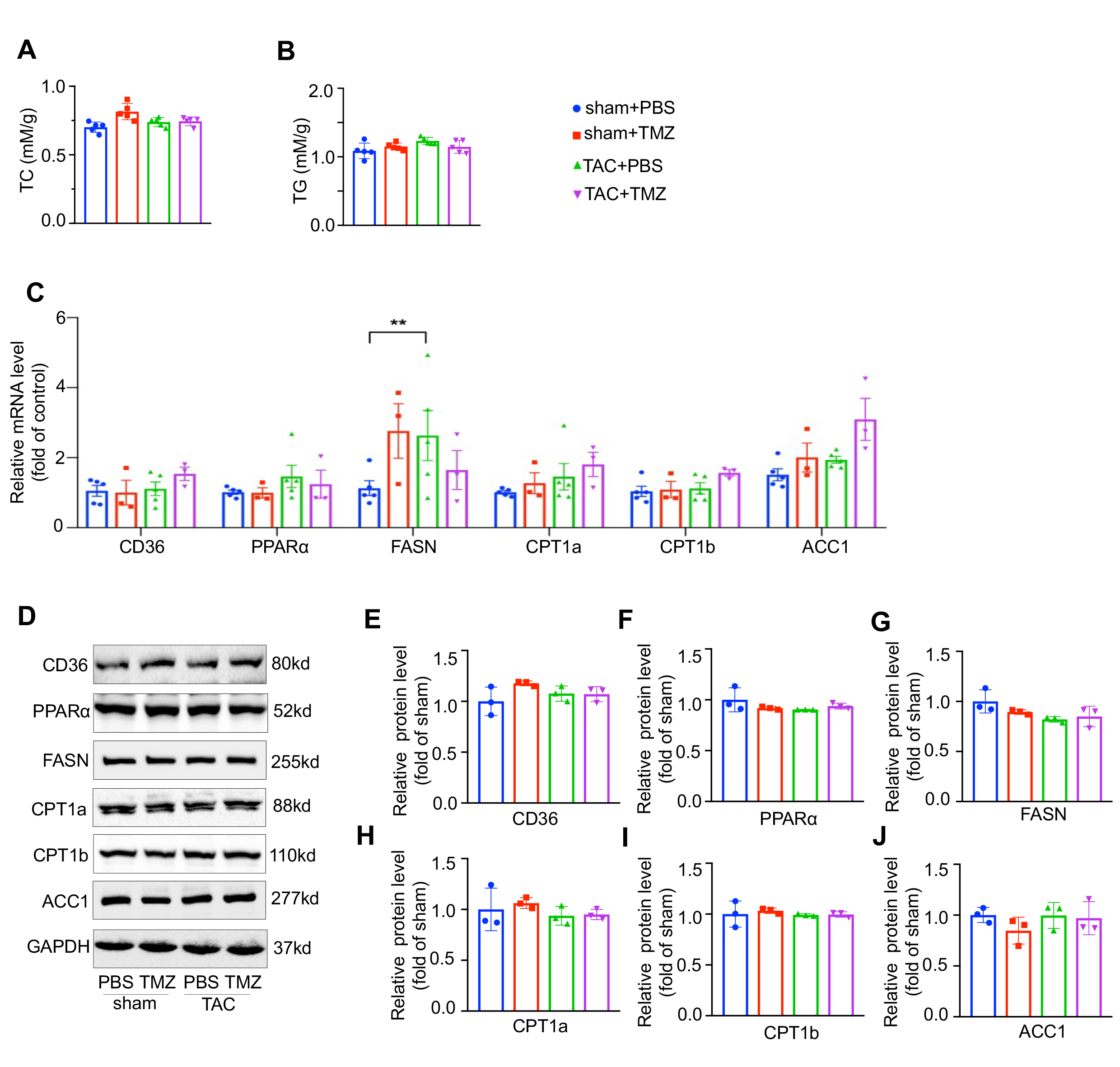

Supplement: Supplementary file 4 [file Image4.JPEG]

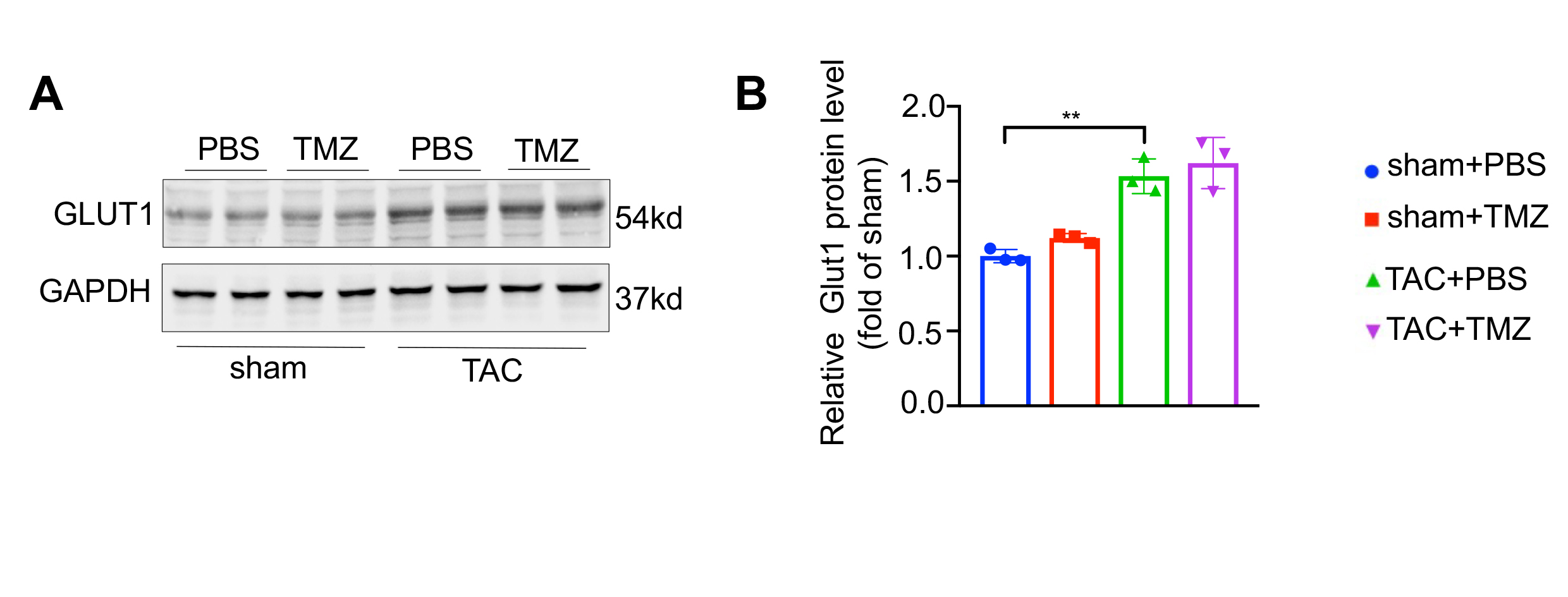

Supplement: Supplementary file 5 [file Image5.JPEG]
